# Modafinil in Post-Traumatic Brain Injury Apathy: A Sleeping Giant?

**DOI:** 10.1192/bjo.2024.75

**Published:** 2024-08-01

**Authors:** Kok Keong Leong, Seth Mensah

**Affiliations:** 1 Aneurin Bevan University Health Board, Blaenau Gwent, United Kingdom; 2 Cardiff and Vale University Health Board, Cardiff, United Kingdom

## Abstract

**Aims:**

Apathy is a complex clinical, neurobehavioural and neurobiological construct that occurs across a range of neuropsychiatric disorders. Apathy is defined as persistent, diminished motivation with impairments in goal-directed behaviour, thought, cognitive activity and emotions. Apathy negatively impacts on participation/engagement in rehabilitation and community reintegration, quality of life, and increased occupational and economic burden on families and traumatic brain injury (TBI) patients. Apathy is among the most common sequelae of TBI, with prevalence estimated to be in excess of 10%, and up to 60% in comorbid depression and apathy.

There is no standard treatment for apathy, although anecdotal evidence suggests that Modafinil may be effective. Current pharmacological management strategies focus on addressing the comorbidities associated with it: e.g. acetylcholinesterase inhibitors to treat both Alzheimer's disease and apathy; dopaminergic agonists for Parkinson's disease and apathy; and antidepressants for depression and apathy.

This literature review will assess the clinical evidence of Modafinil, and recommended use for treating post-TBI apathy.

**Methods:**

An extensive search was conducted in the major databases, PsychInfo, Cochrane, Europe PMC, PubMed, EMBASE and MEDLINE, to evaluate Modafinil treatment for apathy in TBI patients. Additionally, the literature review included extra sources found in the citations. Out of 70 citations, only one was accepted for further analysis. The remaining citations were rejected due to their ineligible abstracts, absence of pharmacological interventions, inclusion of non-TBI apathy and being non-English language articles.

**Results:**

The accepted paper did not meet Level III evidence or better following analysis.

The review however identified case reports suggesting the potential effectiveness of Modafinil in treating post-TBI apathy.

Although the exact mechanism of action of Modafinil remains unclear, it is associated with improvement in working memory, attention and prefrontal-dependent cognitive function. This improvement is linked to elevated levels of extracellular dopamine, norepinephrine, serotonin, glutamate and histamine, as well as decreased GABA levels. Modafinil activates the anterior cingulate cortex, and shows positive correlation with cognitive improvement. Neuroanatomically, there is a strong association between apathy and disruption of the cortico-basal ganglia loop, involving the dorsal anterior cingulate cortex, ventral striatum and connected brain regions. Modafinil possibly has unexplored benefits in improving apathy through activation of the anterior cingulate cortex.

**Conclusion:**

There is limited empirical evidence for effective treatments for post-TBI apathy. This review emphasizes the urgent need for further research that aligns with underlying neuroanatomical pathology in order to determine the most effective psychopharmacological interventions for managing post-TBI apathy.

